# Role of mitophagy in breast cancer: mitophagy-apoptosis balance and reactive oxygen species play determining role

**DOI:** 10.3389/fphys.2025.1716765

**Published:** 2026-01-23

**Authors:** Lung Yiu, Yan Wah Chong, Shuyi Yan, Suk Ying Tsang

**Affiliations:** 1 School of Life Sciences, The Chinese University of Hong Kong, Hong Kong SAR, China; 2 State Key Laboratory of Agrobiotechnology, The Chinese University of Hong Kong, Hong Kong SAR, China; 3 Key Laboratory for Regenerative Medicine, Ministry of Education, The Chinese University of Hong Kong, Hong Kong SAR, China; 4 Institute for Tissue Engineering and Regenerative Medicine, The Chinese University of Hong Kong, Hong Kong SAR, China

**Keywords:** apoptosis reversal, BNIP3, mitophagy, PINK1, reactive oxygen species

## Abstract

This review aims to present a current overview of the role of mitophagy in breast cancer progression, especially from the point of view of when the cancer is in the untreated state or under chemotherapeutic treatment. We aim to explain the apparently contradictory results as reported in numerous studies on the differential role of mitophagy in breast cancer. We propose that different levels of reactive oxygen species (ROS), and the balance between mitophagy and apoptosis under different conditions are the major reasons to explain for the “discrepancy”. If the cancer cells are untreated, a medium level of ROS promotes cancer progression. Mitophagy inhibition, which leave the dysfunctional mitochondria to generate more ROS, would therefore increase cancer progression. On the other hand, if the cancer cells are undergoing chemotherapeutic treatment, the excessively high level of ROS generated would stimulate both mitophagy and apoptosis, where mitophagy would inhibit apoptosis. In this case, inhibiting mitophagy would potentiate apoptosis and therefore enhance treatment effectiveness. The molecular mechanisms underlying the regulation between mitophagy and apoptosis are also discussed in this review. In summary, the review shall provide important insights for the role of mitophagy in breast cancer. It is proposed that the identification of the molecules involved in balancing mitophagy and apoptosis, and combined therapeutic strategies are the key areas for future exploration.

## Breast cancer relapse, breast cancer stem cells (CSCs) and the emergence of breast CSCs by apoptosis reversal

Cancer is one of the top killers in the world. Breast cancer is the most common cancer among women. Among different types of cancers, female breast cancer ranks second in terms of incidence and ranks fourth in terms of mortality when both sexes are counted (Bray et al., 2024). Breast cancer can be classified according to molecular subtypes; the four main molecular subtypes include luminal A [estrogen receptor (ER)^+^ and/or progesterone receptor (PR)^+^, human epidermal growth factor receptor 2 (HER2)^-^, Ki67 ^low/-^], luminal B (ER^+^ and/or PR^+^, HER2^+/−^, Ki67^+^), HER2-enriched (ER^+/−^, PR^+/−^, HER2^+^) and triple negative (ER^−^, PR^−^, HER2^-^) breast cancer (TNBC) (Waks and Winer, 2019).

Currently, there are several treatment options for breast cancer, including surgery, chemotherapy, radiotherapy, and immunotherapy. They are used in treatment for breast cancer patients at different stages and/or of different subtypes (Prat et al., 2015). Surgical removal of the whole tumor is the common treatment method for early stage (stages 0-I) breast cancer patients in many countries. Unfortunately, surgery can only be applied to less than 50% of the breast cancer patients because cancer is usually not diagnosed at an early stage but at the later stage (Prat et al., 2015). In addition, surgery is not useful for patients with a large tumor as surgical removal of large tumor will cause severe bleeding and damage to the organ (Wyld et al., 2015). As a result, surgery is not applied to late stage cancer patients.

Chemotherapy and radiotherapy have been in use since early 20th century (DeVita and Chu, 2008). Chemotherapy is usually applied to breast cancer patients of stage II or higher stages (McDonald et al., 2016). Most drugs used in chemotherapy such as doxorubicin and 5-fluorouracil work by triggering apoptosis in cancer cells (Pistritto et al., 2016); they could reduce the sizes of tumor which can later be removed by surgery. On the other hand, newly developed therapeutic approach immunotherapy works by boosting patients’ immune system so that cancer cells can be recognized and killed more effectively (Keenan and Tolaney, 2020). Unfortunately, cancer relapse was reported in breast cancer patients after immunotherapy (Serrou et al., 1982). Also, the efficacy of chemotherapy is still less than ideal; previous studies reported that for some breast cancer patients treated with chemotherapy, some time after drug withdrawal, breast cancer relapsed (Garcia-Murillas et al., 2015; Garcia-Murillas et al., 2019).

In recent decades, many studies in cancer suggest the emergence of cancer stem cells (CSCs) as the reasons for cancer and/or cancer relapse (Alvarez-Varela et al., 2022; Liu et al., 2020; Zhang et al., 2023). CSCs are the cancer cells found inside tumor which possess some characteristics resembling those of normal stem cells, such as self-renewal and differentiation capability. CSCs contribute to the different processes of cancer progression, including migration, invasion, metastasis, resistance to the immune responses or drugs, and induction of epithelial-to-mesenchymal transition (EMT) (Cabrera et al., 2015; Cazet et al., 2018; O'Brien et al., 2010; Plaks et al., 2015). Since the CSCs cannot be eliminated by chemotherapeutic drugs or immune response due to their resistance properties and/or their ability to escape from immunosurveillance, they can be the driver of cancer relapse under a suitable condition (Lytle et al., 2018). Therefore, to prevent cancer relapse, recent studies have started to explore treatment strategies for eradicating CSCs (Muntimadugu et al., 2016; Scatena et al., 2018; Shigdar et al., 2011).

Importantly, our previous study had shown that when cancer cells were treated with apoptotic inducer and drug withdrawal was done at an inappropriate time, apoptosis reversal would be resulted, leading to a more aggressive population of cancer cells with a higher proportion of CSCs formed (Xu et al., 2018). It is believed that clinically, after treatment with the chemotherapeutic drug, while most of the cancer cells are supposed to be inhibited and/or undergo apoptosis, and that the sizes of tumor are reduced or undetectable; crucially, some of the cancer cells may have been reversed from apoptosis and survive. These reversed cells are stem-like cells with more aggressive behaviors and are resistant to drugs (Xu et al., 2018; Tang et al., 2017), and thereby can account for the cancer relapse. Therefore, apoptosis reversal is believed to be an important process for cancer development post-drug treatment. However, the strategies to sustain apoptosis and/or to inhibit apoptosis reversal so as to completely abolish the emergence of CSCs are needed to be further explored in order to find the possible way to avoid cancer relapse and/or to eradicate cancer. In mitochondria-dependent apoptosis, various stimuli (e.g., oxidative stress, DNA damage) lead to the permeabilization of the mitochondrial outer membrane, a process controlled by the Bcl-2 protein family. This subsequently leads to the release of cytochrome C from the mitochondria into the cytosol, further activating caspases and initiate the apoptotic cascade. All-in-all, mitochondria play an important role in inducing apoptosis (Tait and Green, 2010; Bock and Tait, 2020). We therefore speculate the removal of dysfunctional mitochondria in the process of mitophagy may play a crucial role in apoptosis reversal.

## Mitophagy is unfavorable to proliferation, invasion, metastasis and angiogenesis of untreated breast cancer cells

Dysfunctional mitochondria are detrimental to the cells; they would produce high level of reactive oxygen species (ROS) which would damage the cells by decreasing mitochondrial membrane potential and inducing mitochondrial permeability transition (Lin et al., 2021). Theoretically, mitophagy promotes the degradation of dysfunctional mitochondria and recycles the mitochondrial content to generate healthy mitochondria. For mitophagy to begin, a protein/receptor on the mitochondrial membrane is stimulated; subsequent release of a committed mitophagy signal would then recruits the autophagy machinery for the degradation of mitochondria and the recycling of materials (Ferro et al., 2020; Onishi et al., 2021). Interestingly, not all types of cells tend to repair damaged mitochondria, and the ROS generated by these damaged mitochondria may be beneficial to the cells such as in the case of cancer cells (Chourasia et al., 2015). Chourasia et al. reported that BNIP3, a mitochondrial membrane protein that acts as mitophagy receptor to interact with LC3 of the autophagosome in ubiquitin-independent mitophagy pathway, is deleted in some TNBC patients and predicts poor prognosis (Chourasia et al., 2015). This deletion of BNIP3 was found to lead to excessive ROS production; this in turn led to induction of hypoxia inducible factor 1 (HIF-1) expression, and further promoted cancer cell proliferation, invasion and metastasis (Chourasia et al., 2015). Consistently, another previous study also reported that BNIP3 expression is reduced in invasive breast cancers; tumors which showed loss of BNIP3 expression were reported to have a higher mitotic activity and a higher frequency of lymph node metastasis (Koop et al., 2009). However, although BNIP3 is well-known to be involved in mitophagy, there is no data on the level of mitophagy in patients reported in these studies.

Apart from BNIP3-mediated mitophagy, DRP1/PINK1-mediated mitophagy, the most well-studied ubiquitin-dependent mitophagy pathway, has also been reported to have a role in inhibiting cell growth in breast cancer cells. A previous study has shown that flubendazole, an anthelmintic drug, impaired the permeability of mitochondrial membrane through upregulating of EVA-1 homolog A, and further increased the expression of DRP1 and PINK1. This subsequently led to excessive mitophagy, which in turn inhibit the proliferation and migration of breast cancer cells (Zhen et al., 2022).

Over half of the woman who have mutation either on BRCA1 or BRCA2 genes will develop breast cancer in their lives (Premnath and O'Reilly, 2020). Interestingly, a previous study reported that the PARP inhibitor AZD2281 (Olaparib) in BRCA1 and BRCA2 mutant breast cancer cells induced mitophagy and led to growth inhibition (Arun et al., 2015). However, whether mitophagy and growth inhibition occur in parallel or in the same pathway remains to be investigated. On the other hand, another previous study has shown that overexpression of PINK1 enhanced CCCP-induced BRCA1 degradation and suppressed the proliferation of breast cancer (Miyahara et al., 2021).

Suppression of mitophagy was also found to promote breast cancer bone metastasis under hypoxia condition. Depletion of ULK3, a key protein for mitophagy induction in response to hypoxia, was found to attenuate mitophagy and lead to ROS accumulation; this high level of ROS in turn activated NLRP3 inflammasome, causing the abnormal secretion of cytokines which is beneficial to the differentiation and maturation of osteoclasts and ultimately bone metastasis of breast cancer (Deng et al., 2021). In addition, enhanced mitophagy has also been reported to inhibit tumor angiogenesis. Decorin, a proteoglycan expressed in the stroma of various cancer, has been reported to enhance mitophagy in a PGC-1α- and mitostatin-dependent manner; its application was shown to suppress tumor angiogenesis by decreasing the production of vascular endothelial growth factor A in breast cancer cells (Neill et al., 2014).

Another study suggested that a phenanthroline copper (II) complex CPT8, on one hand, induced mitophagy in breast cancer cells through activation of PINK1/Parkin and BNIP3 pathways; on the other hand, CPT8 suppressed the expression of vascular endothelial cadherin and matrix metalloproteinases MMP2 and MMP9, key factors for tumor angiogenesis (Zhen et al., 2022). However, it is undetermined whether mitophagy and the anti-angiogenesis effect occur in parallel or in the same pathway. Also, CPT8 was found to enhance both the apoptosis and mitophagy. It is unclear whether the mitophagy will be acting against apoptosis or promoting apoptosis.

Furthermore, drug resistance and immunotherapy resistance are the key reasons for the low effectiveness of chemo- and immuno-therapy. A previous study revealed that promoting ATAD3A-PINK1-mediated mitophagy pathway redirected PD-L1 to mitochondria for degradation and therefore could be a potential strategy of overcoming chemoimmunotherapy resistance (Xie et al., 2023). Another study reported that simultaneous treatment by chemotherapy and MEK inhibition by Trametinib promoted mitophagy and allowed the cancer cells to escape from chemoimmunotherapy resistance (Limagne et al., 2022). Thus, these studies suggested that inducing mitophagy would have a positive effect on the effectiveness of chemoimmunotherapeutic treatments.

Altogether, it appears that mitophagy induction in breast cancer cells would lead to inhibition of tumor growth and tumor progression, especially when the cells are untreated or are not treated with chemotherapy alone (Table 1).

**TABLE 1:** Table summarizing studies documenting the positive or the negative role of mitophagy in breast cancer progression and the involvement of ROS in the process.

Cancer type	*In vitro*/*In vivo* study	Properties measured	ROS measured? What has been found?	Citation
Mitophagy is unfavorable to cancer progression				
Breast cancer	*In vitro*	Proliferation, Migration	Yes. Low ROS is favorable. BNIP3 deletion → ROS → HIF-1 stabilization → cancer progression	Chourasia et al. (2015)
Breast cancer	*In vitro*	Proliferation, Migration, Angiogenesis	Not specified. Flubendazole impairs mitochondrial membrane → mitophagy → inhibits proliferation/migration. CPT8 induced mitophagy and suppressed angiogenesis	Zhen et al. (2022)
Breast cancer	*In vitro*	Proliferation	Not specified. PARP inhibitor induced mitophagy and growth inhibition (parallel or causal link unclear)	Arun et al. (2015)
Breast cancer	*In vitro*	Proliferation	Not specified. PINK1 overexpression suppressed proliferation	Miyahara et al. (2021)
Breast cancer	*In vitro and* * in vivo*	Metastasis (Bone)	Yes. ULK3 depletion → attenuated mitophagy → ROS accumulation → NLRP3 activation → bone metastasis	Deng et al. (2021)
Breast cancer	*In vitro*	Angiogenesis	Not specified. Decorin enhanced mitophagy → decreased VEGF → suppressed angiogenesis	Neill et al. (2014)
Breast cancer	*In vitro *and *in vivo*	Drug Resistance	Not specified. Promoting mitophagy degraded mitochondrial PD-L1 → overcame resistance	Xie et al. (2023)
Mitophagy is favorable to cancer progression				
Breast cancer	*In vitro and* *in vivo*	Tumor Growth, Drug Resistance	Not specified. MUC1-induced mitophagy → chemoresistance and CSC appearance	Li et al. (2022), Jin et al. (2017), Lv et al. (2019)
Breast cancer	*In vivo*	Tumor Growth, Drug Efficacy	Yes. Mitophagy inhibitor (chloroquine) + chemodrug → enhanced ROS-CaMKII/Drp1 signaling → stronger suppression	Hu et al. (2019)
Breast cancer	*In vitro*	Cell Death, Drug Sensitivity	Yes. B5G1-induced ROS activates both apoptosis and mitophagy. Inhibiting mitophagy → enhanced apoptosis	Yao et al. (2019)
Breast cancer	*In vitro*	Apoptosis, Drug Sensitivity	Not specified. Inhibiting mitophagy (liensinine) → enhanced doxorubicin-induced apoptosis	Zhou et al. (2015)
Breast cancer	*In vitro*	Apoptosis, Cell Survival	Yes. Warangalone induced mitophagy and apoptosis. Mitophagy protected against apoptosis	Mao et al. (2021)
Breast cancer	*In vitro*	Apoptosis, Cell Survival	Not specified. ARIH1 activated PINK1/mitophagy → cell survival. ARIH1 knockdown → increased apoptosis	Villa et al. (2017)
Breast cancer	*In vitro*	Apoptosis, Drug Sensitivity	Not specified. PINK1 knockdown → sensitized cells to paclitaxel-induced apoptosis	MacKeigan et al. (2005)
Breast cancer	*In vitro*	Apoptosis, Drug Sensitivity	Not specified. miRNA-218-5p (inhibited Parkin/mitophagy) → sensitized to doxorubicin	Naso et al. (2024)
Breast cancer (CSCs)	*In vitro*	CSC Maintenance	Not specified. p52-ZER6 → pro-survival mitophagy → maintained CSCs	Li et al. (2024)
Breast cancer	*In vitro* and *in vivo*	Tumor Growth, Drug Resistance	Not specified. MUC1-induced mitophagy → chemoresistance and CSC appearance	Li et al. (2022)

## Mitophagy is a favorable process to rescue cancer cells under chemotherapeutic drug treatment

### Mitophagy is favorable for the survival of cancer cells upon chemotherapeutic drug treatment

A number of previous reports have revealed that co-application of chemotherapeutic drugs and mitophagy inhibitors led to a strong suppressive effect on cancer growth than application of chemotherapeutic drugs alone (Hu et al., 2019; Yao et al., 2019; Zhou et al., 2015). For instance, a stronger suppression of tumor growth was observed in xenograft mice treated with a combination of chemotherapeutic drug (isorhamnetin) and mitophagy inhibitor (chloroquine) when compared with xenograft mice treated with isorhamnetin alone. The stronger suppression was found to be attributed to the enhanced ROS-mediating CaMKII/Drp1 signaling (Hu et al., 2019). Besides, inhibition of mitophagy using mdivi-1 and bafilomycin A1 was found to promote B5G1 (a new derivative of betulinic acid)-induced cell death and increase the effectiveness of chemotherapy (Yao et al., 2019). It is reasoned that some chemotherapeutic drugs would cause mitochondrial dysfunction and enhance ROS production; mitophagy is a protective process in the cancer cells to remove dysfunctional mitochondria and is thereby an adaptation to chemotherapy drug treatment. Similarly, inhibiting mitophagy using liensinine was found to enhance doxorubicin-mediated apoptosis by triggering mitochondrial fission through dephosphorylation and mitochondrial translocation of DRP1 (Zhou et al., 2015). Therefore, this study revealed that mitophagy inhibitor liensinine could increase the sensitivity of breast cancer cells to chemotherapy by regulating the activity and subcellular localization of DRP1.

Mechanistically, some treatments that trigger apoptosis simultaneously stimulate mitophagy, where mitophagy acts against apoptosis during upon chemotherapeutic drug treatment; stimulating apoptosis while inhibiting mitophagy can thereby enhance the effectiveness of chemotherapeutic drugs. A previous study reported that after inducing mitochondrial depolarization using chemotherapeutic drug or CCCP, E3 ubiquitin ligase ARIH1 would activate PINK1 and trigger PINK1-mediated mitophagy and protect cancer cells from these drugs to survive; on the other hand, knockdown of ARIH1 increased the apoptosis of cancer cells upon treatment with these drugs (Villa et al., 2017). Consistently with these studies, knockdown of PINK1 was found to sensitize BT474 (a HER2^+^ breast cancer cell line) to apoptosis induced by paclitaxel, a common chemotherapeutic drug (MacKeigan et al., 2005). Similarly, knocking down Parkin by miRNA-218-5p, which was sufficient to inhibit doxorubicin-mediated mitophagy, sensitized breast cancer cells to doxorubicin (Naso et al., 2024). Also, for other drugs that have shown efficacy in inducing apoptosis in breast cancer cells, they have at the same time been shown to induce mitophagy which attenuated apoptosis (Mao et al., 2021; Si et al., 2020; Pu et al., 2025); inhibiting mitophagy in those cases therefore was shown to augment apoptosis.

Altogether, some drugs that would induce apoptosis in breast cancer cells would trigger mitophagy simultaneously; this mitophagy is acting against apoptosis. Inhibiting mitophagy while applying the chemotherapeutic drugs can therefore enhance the efficacy of breast cancer treatment. The molecular mechanisms underlying the inhibitory effect of mitophagy to apoptosis will be further discussed in this article.

### Mitophagy is favorable for the appearance and/or survival of CSCs

A recent study reported that p52-ZER6, a zinc-finger protein, was crucial for CSC maintenance (Li et al., 2024). P52-ZER6 works by regulating the transcription of insulin-like growth factor 1 receptor which promotes pro-survival mitophagy and helps the maintenance of CSCs. On the other hand, some studies highlighted that the chemoresistance of tumor is related to CSCs, where mitophagy may contribute to appearance of CSCs after chemotherapy (Zhao, 2016). Consistently, some recent studies have suggested that modulation of mitophagy contributed to progression of CSCs (Xie et al., 2015). For example, PINK1 activated p53 in hepatic cancer through phosphorylation of p53 on the mitochondrial membrane and mediated mitophagy-dependent p53 degradation, favouring NANOG expression and subsequently the proliferation of hepatic CSCs (Nazio et al., 2019). In addition, oncoprotein mucin 1 (MUC1), which was known to target mitochondria to attenuate drug-induced apoptosis, was found to induce the degradation of ATPase family AAA domain-containing 3A (ATAD3A) and thereby protect PINK1 from being cleaved by ATAD3A. This enhanced MUC1-induced mitophagy was found to be positively associated with the tumorigenicity of cancer (Li et al., 2022). In addition, several studies have suggested that MUC1-mediated mitophagy contributes to the development of chemotherapy resistance and the appearance of CSCs in breast cancer (Jin et al., 2017; Lv et al., 2019) (Table 1). Altogether, it would be interesting to investigate if mitophagy is beneficial to apoptosis reversal in breast cancer cells and contributes to the appearance of breast CSCs.

### Molecular mechanisms underlying the inhibitory effect of mitophagy on apoptosis

#### Involvement of PINK1/Parkin pathway

PINK1 has been reported to inhibit apoptosis through direct protein-protein interaction with Bcl-2 family proteins. At outer mitochondrial membrane (OMM), PINK1 phosphorylates Bcl-xL to hinder its pro-apoptotic cleavage and phosphorylates pro-apoptotic BAD to reduce its mitochondrial localization (Arena et al., 2013; Wan et al., 2018). Increased proportion of anti-apoptotic Bcl-2 family member proteins on the OMM plays a vital role in OMM integrity through inhibition of pore formation, thereby suppressing the release of cytochrome c and downstream activation of caspase (Borras et al., 2020). Since Bcl-xL was also reported to allow the diffusion of metabolites across the OMM by inhibiting the closure of voltage-dependent anion channel (VDAC) and thereby sustaining the permeability to ADP (without inducing the loss of cytochrome c) (Vander Heiden et al., 2001), increased Bcl-xL on the OMM caused by the activity of PINK1 would attenuate apoptosis. Bcl-2-interacting protein-1 (Beclin1) is a protein involves in autophagosome assembly. A previous study has found that caspase 3-mediated Beclin1 cleavage would not only impair mitophagy, but would also generate a Beclin-1-C fragment that can enhance cytochrome c release, thus establishing a positive feedback loop that promotes apoptosis (Wirawan et al., 2010). Interestingly, PINK1 has been shown to directly interact with Beclin 1 and inhibit the pro-apoptotic cleavage of Beclin1 (Brunelli et al., 2022).

**FIGURE 1:**
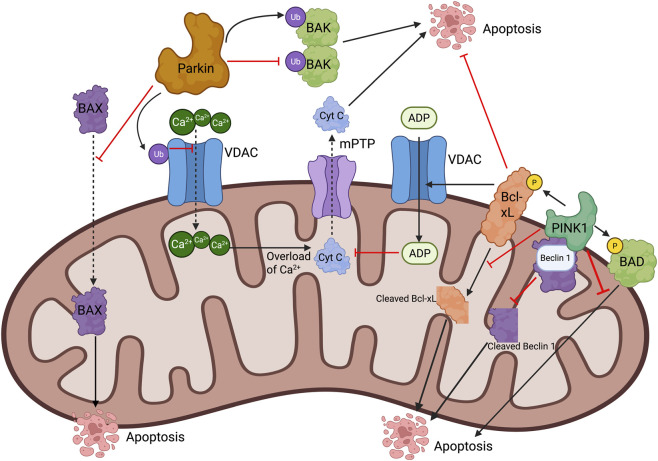
Molecular mechanisms underlying the inhibitory effect of PINK1/Parkin-mediated mitophagy on apoptosis. Details are elaborated in the text.

The E3 ubiquitin ligase Parkin has also been found to play a key role in apoptosis inhibition. A previous study has found that Parkin-mediated ubiquitination of pro-apoptotic Bcl-2 family protein BAK at its hydrophobic groove reduced the homo-dimerization activity of BAK (Bernardini et al., 2019). Parkin has also been shown to reduce the mitochondrial localization of pro-apoptotic BAX independent of ubiquitination (Bernardini et al., 2019). VDAC is another substrate of Parkin (Ham et al., 2020). Mono-ubiquitination of VDAC is a protective mechanism against mitochondrial calcium overload and the opening of mitochondrial permeability transition pore (mPTP) (Ham et al., 2020). Poly-ubiquitination of VDAC can recruit autophagy cargo receptors (Geisler et al., 2010). Therefore, either PINK1/Parkin inhibits apoptosis by maintaining mitochondrial health through mitophagy or directly counteracts the apoptosis signaling by inhibiting pro-apoptotic proteins and/or sustaining the function of anti-apoptotic proteins; these mechanisms help cancer cells to escape from apoptosis. Figure 1 summarizes how PINK1 and Parkin can act against apoptosis at molecular level.

#### Involvement of BNIP3/NIX pathway

In lung adenocarcinoma, cisplatin was reported to induce elevated apoptosis in the BNIP3-deficient mutant compared to wildtype (Q et al., 2010). In ovarian carcinoma and osteosarcoma, cisplatin resistance was found to be associated with high BNIP3 expression (Ma et al., 2017). A previous study has highlighted the anti-apoptotic effect of BNIP3 at the molecular level. The active BNIP3 (with LIR phosphorylation) was found to reduce cellular cytochrome c (Billen et al., 2008). The same paper also has reported a landmark discovery: In MCF-7 with stable expression of mitochondrial cytochrome c, overexpression of Bcl-xL blocks OMM permeabilization and thus inhibits mitophagy in the actively respiring mitochondria; however, co-expression of Bcl-xL and BNIP3 remarkably induces mitophagy in those mitochondria (Billen et al., 2008). In this process, BNIP3 seems to perform mitophagic removal in advance of the release of pro-apoptotic signal (cytochrome c) release. Meanwhile, studies have found that genetic knockdown/knockout of BNIP3 induced the mitophagy defective phenotype: the accumulation of dysfunctional mitochondria impaired mitochondrial respiration, destabilized electron transport chain, and led to robust mROS generation, which are sufficient to stimulate downstream events including cytochrome c release, caspase activation, PARP cleavage (Q et al., 2010; Vande Velde et al., 2000). Overall, these studies suggest that BNIP3 acts as an inhibitor of apoptosis and rescues the cancer cells from apoptosis.

## Discussion

### Relationship between apoptosis and mitophagy: mitophagy may act as counteracting force of apoptosis in apoptotic cancer cells

While some reports indicated that apoptosis or mitophagy alone in most situations may be unfavorable to the survival of cancer (Chourasia et al., 2015; Zhen et al., 2022; Arun et al., 2015; Deng et al., 2021) and that triggering/enhancing the process is a strategy for cancer treatment, there are still numerous cases documenting cancer relapse following the treatment. Interestingly, ample evidence has shown that when cancer cells are undergoing apoptosis, mitophagy is activated and takes a role in rescuing the cells by recycling the damaged mitochondria. Indeed, a number of publications have highlighted the important role of mitophagy on the development of radio- or chemo-resistance of different cancer types through PINK1/Parkin-, BNIP3/NIX- and/or FUNDC1-mediated pathways (Chang et al., 2019; Genovese et al., 2021). However, whether mitophagy plays a role in apoptosis reversal and cancer relapse remains to be determined. In summary, mitophagy may not be beneficial to the survival of cancer cells, but in some cases when apoptosis is induced, mitophagy is the counteracting force of apoptosis and helps to rescue the cancer cells.

### Possible model for the inhibitory effect of mitophagy on apoptosis

A previous study has shown that mitophagy was upregulated in response to mitochondrial stress and prevented mitochondrial-mediated apoptosis. B5G1, a betulinic acid analog, was found to exhibit mitochondrial toxicity in multidrug-resistant cancer. This study specifically found that B5G1 treatment induced cytochrome c release, cleavage of caspase-9, caspase 3 and PARP but not caspase-8, suggesting that apoptosis occurred only through the intrinsic apoptosis pathway. The time points of mitophagy and apoptosis initiation were also revealed in this study. B5G1-induced ROS production was found to serve as the activator of both mitophagy and apoptosis (Yao et al., 2019). We suggest a possible model to explain the sequence of events and the mode of interaction between the mitophagy and apoptotic pathways: (i) stress induces ROS production which trigger both mitophagy and mitochondrial apoptosis; (ii) mitophagy and apoptosis compete with each other: mitophagy inhibits apoptosis by the removal of damaged mitochondria to inhibit the release of pro-apoptotic factors (e.g., cytochrome or by direct crosstalk with apoptotic pathways) or by the molecular mechanisms as discussed above (under the section of “Molecular mechanisms underlying the inhibitory effect of mitophagy on apoptosis”); (iii) mitophagy has a limited capacity to inhibit apoptosis; if overwhelming stress or loss of mitochondria persists, this will tilt the balance in favor of apoptosis; (iv) apoptosis further weakens mitophagy, thus constituting a positive feedback for apoptosis.

Intriguingly, the degree of influence of mitophagy on antagonizing apoptosis induced by pro-apoptotic signals may depend on the “tolerance” of the cells to pro-apoptotic signals. One study compared the phenotype of cisplatin-sensitive and cisplatin-resistant ovarian carcinoma cell lines. The resistant cell line was found to exhibit a low level of p53 and a fragmented phenotype of mitochondria (Vianello et al., 2022). Low p53 levels could contribute to a less sensitive intrinsic apoptotic pathway (Aubrey et al., 2018). Mitochondria fragmentation caused by enhanced mitophagy is considered an early hallmark of apoptosis (Vianello et al., 2022), but apoptosis is curtailed due to known mechanisms. We speculate that the insensitivity of cancer cells to perceive death “buys time” for mitophagy to eliminate pro-apoptotic stress. In addition, if the apoptotic inducer is removed at an inappropriate time when the apoptotic processes in some cancer cells have not been completed, apoptosis reversal would be resulted.

### Possible reasons for the different outcomes of mitophagy on cancer progression: level of ROS

Induction of mitophagy in chemodrug-resistance or chemodrug-sensitive cancer cells leads to different outcomes. For example, in ovarian carcinoma, one study reported that BNIP3 increased cisplatin-induced apoptosis in cisplatin-sensitive cells, while in another study, BNIP3-mediated mitophagy contributed to cisplatin resistance (Vianello et al., 2022; Jia et al., 2020). In the first study, the cisplatin treatment caused significant increase of BNIP3 expression, and knockdown of BNIP3 reduced cisplatin cytotoxicity and partially alleviated cisplatin-induced apoptosis (Jia et al., 2020). In the second study, compared with the cisplatin-sensitive cell line, the resistant clones exhibited a fragmented phenotype of mitochondria, a higher expression of fission proteins, a higher autophagic flux after starvation, a higher expression of BNIP3, and a lower mitochondrial mass (Vianello et al., 2022). Indeed, rates of mitophagy were also increased. Importantly, silencing BNIP3 or pharmacological inhibition of autophagosome formation re-sensitizes these cells to cisplatin, suggesting inhibiting BNIP3 and/or mitophagy together with chemotherapeutic drug application can be used as a therapeutic strategy to treat cancer.

We speculate that one of the reasons of observed oppose effects of mitophagy on cancer is attributed to the level of ROS generated by different cancer cells. The dependence of cancer cells on the OXPHOS pathway for energy production affects cancer’s vulnerability to mitophagy inhibition. Sorafenib (SFB) is a mitochondria-targeting drug that inhibits OXPHOS, reduces ATP production and enhances ROS production. A previous study suggested that the negativity of SFB treatment for certain types of cancers is attributed to the effects of metabolic reprogramming on aerobic glycolysis (Tesori et al., 2015). Along this line, we speculate that if the cancer cells depend more on glycolysis but less on mitochondria for energy production, inhibiting removal of dysfunctional mitochondria by mitophagy inhibition will generate ROS at a level that is favorable for cancer progression. On the other hand, if the cancer cells rely more on mitochondria for energy production, inhibiting removal of dysfunctional mitochondria will lead to massive ROS generation which is unfavorable to cancer progression.

**FIGURE 2:**
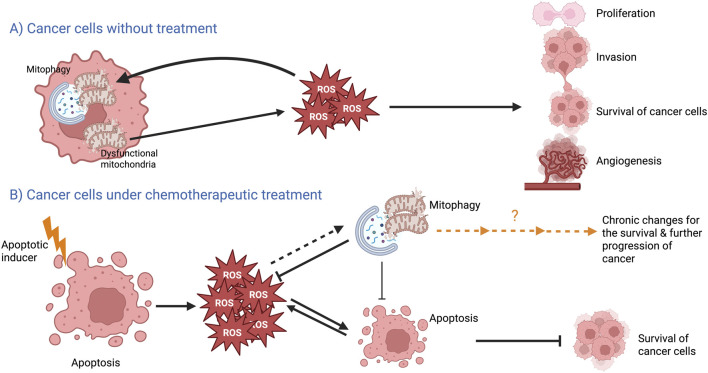
Proposed models for the role of mitophagy in **(A)** untreated breast cancer cells and **(B)** breast cancer cells under chemotherapeutic treatment. Details are elaborated in the text.

The proposed models for the differential effects of mitophagy on cancer progression are presented in Figure 2.

### Unanswered questions

In the future, we suggest investigating the suitable time of inhibition of mitophagy to synergize the effect of chemotherapeutic drugs to induce apoptosis. Also, we suggest investigating the signature event that represents the turning points of the competition between mitophagy and apoptosis. In addition, although the molecular mechanisms underlying how mitophagy can counteract apoptosis in other cell types are starting to be elucidated, the role of mitophagy/mitophagy-related proteins on cancer relapse remains to be investigated.

## Conclusion

Altogether, mitophagy may help prevent cancer progression by decreasing mitochondrial ROS production. On the other hand, when cancer cells are undergoing cytotoxic stress such as those caused by chemotherapeutic treatment and immunotherapy, mitophagy is activated to degrade damaged mitochondria and reduce mitochondrial ROS and thereby is acting in an antagonistic way to protect cancer cells from apoptosis (Xie et al., 2023). In the latter case, co-treatment of cancer cells with chemotherapeutic drugs and mitophagy inhibitor may exert a much better efficacy in cancer treatment and may help eradicate cancer. In the near future, detailed studies are urgently needed to investigate the relationship between apoptosis and mitophagy, and whether mitophagy could be a novel target for treatment of breast cancer. In fact, one of the main reasons for the high mortality rate of breast cancer is that after chemotherapeutic drug withdrawal, cancer relapse occurs. More investigations on mitophagy and breast cancer relapse are needed in order to develop a highly effective targeted therapy for breast cancer patients.
